# Endophytes: A Treasure House of Bioactive Compounds of Medicinal Importance

**DOI:** 10.3389/fmicb.2016.01538

**Published:** 2016-09-29

**Authors:** Sushanto Gouda, Gitishree Das, Sandeep K. Sen, Han-Seung Shin, Jayanta Kumar Patra

**Affiliations:** ^1^AMITY Institute of Wildlife Science, NoidaIndia; ^2^Research Institute of Biotechnology and Medical Converged Science, Dongguk University-Seoul, GoyangSouth Korea; ^3^Department of Biotechnology and Medical Engineering, National Institute of Technology Rourkela, RourkelaIndia; ^4^Department of Food Science and Biotechnology, Dongguk University-Seoul, GoyangSouth Korea

**Keywords:** bioactive compounds, endophytes, foodborne diseases, secondary metabolites

## Abstract

Endophytes are an endosymbiotic group of microorganisms that colonize in plants and microbes that can be readily isolated from any microbial or plant growth medium. They act as reservoirs of novel bioactive secondary metabolites, such as alkaloids, phenolic acids, quinones, steroids, saponins, tannins, and terpenoids that serve as a potential candidate for antimicrobial, anti-insect, anticancer and many more properties. While plant sources are being extensively explored for new chemical entities for therapeutic purposes, endophytic microbes also constitute an important source for drug discovery. This review aims to comprehend the contribution and uses of endophytes as an impending source of drugs against various forms of diseases and other possible medicinal use.

## Introduction

Plants have served as a source of medicinal bioactive compounds against numerous forms of ailments for centuries. Ironically, in recent years, microorganisms associated with plants rather than plants themselves have proved to offer material and products with high therapeutic potential (Subbulakshmi et al., 2012). Endophytes are an endosymbiotic group of microorganisms – often bacteria or fungi – that colonize the inter- and/or intracellular locations of plants (Pimentel et al., 2011; Singh and Dubey, 2015). For these organisms, all or part of their life cycle occurs within their hosts, without causing any apparent symptoms of disease. They are ubiquitous in nature and exhibit complex interactions with their hosts, which involve mutualism, antagonism and rarely parasitism (Nair and Padmavathy, 2014). Endophytes are known to enhance host growth and nutrient gain. They may improve the plant’s ability to tolerate various types of abiotic and biotic stresses, and enhance the resistance of plants to insects and pests. They produce phytohormones and other bioactive compounds of biotechnological interest (enzymes and pharmaceutical drugs) (Joseph and Priya, 2011; Parthasarathi et al., 2012).

Researchers have indicated the presence of one or more types of endophytes in every single plant studied to date (Strobel and Daisy, 2003). Endophytes can colonize in the stem, roots, petioles, leaf segments, inflorescences of weeds, fruit, buds, seeds and also dead and hollow hyaline cells of plants (Hata and Sone, 2008; Specian et al., 2012; Stępniewska and Kuzniar, 2013). The population of endophytes in a plant species is highly variable and depends on various components, such as host species, host developmental stage, inoculum density and environmental condition (Dudeja and Giri, 2014).

However, very few studies have exploited these symbiotic groups of organisms and their bioactive metabolites. For the past few decades, it has become evident that the discovery rate of active novel chemical entities is declining. While plant sources are being extensively explored for the discovery of new chemical entities for various therapeutic purposes, endophytic microorganisms play an important role in this search for natural bioactive compounds, with potential use in the health sector and in drug discovery (Lam, 2007). This review highlights the various sources of endophytes, their secondary metabolites and role as source of drugs. Such studies may improve the understanding of endophytes and address the need for new and useful compounds necessary to combat various pathogens associated with human health and other possible medicinal uses.

## What is an Endophyte?

Numerous surveys, mostly on the pathogenicity interactions between plants and microorganisms associated with them, have been accomplished. However, after several explanations and studies on the role of microbial diversity associated with various plant species, it was assumed that only a small fraction of the microbes interacting with the plant are pathogenic in nature (Andreote et al., 2014). Most of the microorganisms that inhabit plants play a major role in the plant’s health and development, although, sometimes they are neutral (Mendes et al., 2013; Philippot et al., 2013). It is considered that a single plant species could possess thousands of microbes, categorized as epiphytes (microbial inhabitants of the rhizosphere and phyllosphere; those near or on plant tissue) or endophytes (microbes residing within plant tissues in leaves, roots or stems), depending on their area of colonization in the plant species (Oldroyd et al., 2011; Turner et al., 2013; Andreote et al., 2014).

Apart from the disease-causing microorganisms, the presence of other (non-pathogenic) organisms inside the plants was first pronounced by De Bary (1866), who detected the presence of microbial cells in the microscopically analyzed plant tissues. However, this observation continued to be unexplored until the definition of endophytes came into existence toward the end of last century. De Bary (1866) provided the first definition of an endophyte, as “*any organism that grows within plant tissues are termed as endophytes*,” however, the definition continues to change as per various researchers (Wilson, 1995; Hallmann et al., 1997; Bacon and White, 2000). Petrini (1991) provided the most suitable definition for endophytes, which means any organism that at some part of its life cycle, colonizes the internal plant tissues without causing any type of harm to the host plant. Furthermore, due to extensive studies of these groups of microorganisms, the endophytic communities have been divided into different subgroups, such as ‘obligate’ or ‘facultative,’ which are associated with all types of plants (Rosenblueth and Martínez-Romero, 2006). Endophytes that depend on the metabolism of plants for survival, being spread amongst plants by the activity of different types of vectors or by vertical transmission, are termed obligate endophytes (Hardoim et al., 2008). Whereas, the facultative endophytes are those that live outside the host body during a certain stage of their life cycle and are mostly associated with plants from its neighboring soil environment and atmosphere (Abreu-Tarazi et al., 2010).

Numerous attempts have been made during recent years to discover the origin of endophytic organisms in different species (Hallmann et al., 1997; Mitter et al., 2013). Initially, researchers choose the rhizosphere or the seed-born microbial communities as the major sources of endophytes. Normally, the specific endophytes interaction with various plants and evidence of their strategy of existence and transmission is provided by their genome organization (Andreote et al., 2014). Researchers have reviewed the genome sizes and origins of endophytes by correlating the genome size with the bacterial lifestyle (Dini-Andreote et al., 2012). As per many researchers, the definition of endophytes could be suitable for the hypothesis that they live inside the plant species, where the environment is more stable compared to the soil, where the plant grows. However, there are also some endophytes that only appear in the plant during part of their lifecycle. Thus, the endophytic community is made up of organisms from distinct origins, with those with larger genomes likely to live in variable environments, such as soils, while those with smaller genomes are likely to exist in the stable environment and are vertically transmitted (Mitter et al., 2013).

## Types of Endophytes

Endophytes are associated with plants in various forms, including bacteria (actinomycetes or mycoplasma) or fungi that have been colonized inside the plant tissues. More than 200 genera from 16 phyla of bacterial species have been reported to be associated with endophytes and among them, most of the species belong to the phyla Actinobacteria, Proteobacteria, and Firmicutes (Golinska et al., 2015). The diversity of endophytic bacteria ranges from gram-positive to gram-negative bacteria, such as *Achromobacter, Acinetobacter, Agrobacterium, Bacillus, Brevibacterium, Microbacterium, Pseudomonas, Xanthomonas* etc. (Sun et al., 2013). Bacterial endophytes are diverse in nature and are known to produce different bioactive metabolites that act as antimicrobial and anticancer compounds, for example, with 76% of them reported from the single genus, *Streptomyces* (Berdy, 2012).

Actinomycetes are prokaryotic microorganisms that belong to the phylum Actinobacteria and possess mycelium like fungus and forms spores (Chaudhary et al., 2013; Barka et al., 2016). Traditionally, these actinomycetes were considered transitional forms between the fungi and bacteria (Barka et al., 2016). However, the comparison of actinomycetes to fungi is only superficial because most of their properties are similar to those of bacteria but unlike bacterial cells, the cells of actinomycetes are thin with a chromosome that is organized in a prokaryotic nucleoid and a peptidoglycan cell wall. Endophytic actinomycetes are known to produce various chemical entities with unique structures of considerable medicinal importance (Gayathri and Muralikrishnan, 2013; Singh and Dubey, 2015). Many antimicrobial compounds have been reported from various types of endophytic actinomycetes. *Streptomyces* is one of the dominant genera, which is most commonly isolated as endophytic actinomycetes (Zhao K. et al., 2011; Golinska et al., 2015). Compounds of biological interest isolated from *Streptomyces* sp. include munumbicins (A and B), naphthomycin (A and K), clethramycin, coronamycin, cedarmycin (A and B), saadamycin, and kakadumycins. Other active compounds isolated from actinomycetes are paclitaxel extracted from *Kitasatospora* sp. associated with *Taxus baccata*, and tyrosol from *Emblica officinalis*, which is suggested to inhibit food-borne microbes (Zhao K. et al., 2011; Gangwar et al., 2014; Golinska et al., 2015).

Mycoplasma species are also reported as plant endophytes. Endophytic mycoplasma species were conveyed to be in a symbiotic relationship with some red algae, such as *Bryopsis pennata*, *B. hypnoides* and also in Arcobacte (Hollants et al., 2011). However, there is no confirmed evidence of its uses, the source of extraction or use against foodborne diseases or other pathogens.

Fungi are a heterotrophic group of organisms with various life cycles that include symbiotic relationships with a wide variety of autotrophic organisms (Dayle et al., 2001). Endophytic fungi have been classified into two broad groups based on their phylogeny and life history traits. These include the clavicipitaceous, which infect some grasses confined to cool regions and the non-clavicipitaceous endophytes, which are from asymptomatic tissues of non-vascular plants, ferns and allies, conifers and angiosperms and are limited to the Ascomycota or Basidiomycota group (Jalgaonwala et al., 2011; Bhardwaj and Agrawal, 2014). Endophytic fungi produce some of the most broadly used antibiotic and anticancer drugs. Penicillenols, extracted from *Penicillium* sp., is cytotoxic to numerous cell lines. Taxol, isolated from *Taxomyces andreanae*, is the most effective and successful anticancer drug extracted from endophytic fungi to date. Clavatol (*Torreya mairei*), sordaricin (*Fusarium* sp.), jesterone (*Pestalotiopsis jesteri*), and javanicin (*Chloridium* sp.) are all known to possess strong antibacterial and antifungal properties against numerous foodborne infectious agents (Jalgaonwala et al., 2011). Pestacin, isolated from *P. microspora*, has excellent antioxidant properties.

## Isolation and Identification of Endophytes from Different Sources

Though the endophytes were overlooked for a long time and are considered as pathogen-causing contaminations, many that inhabit inside the plants are often recognized as symbionts, with a distinctive and cherished interaction with the plants with whom they grow (Mitter et al., 2013; Berg et al., 2014). Recent studies have confirmed the occurrence of endophytes by various cultivation-independent assays and by fluorescence *in situ* hybridization-confocal laser scanning microscopy studies (Mitter et al., 2013; Berg et al., 2014). Cultivation-based techniques, use the recovery and testing of isolates, whereas cultivation-independent techniques screen for variations in the total endophytic communities (Menpara and Chanda, 2013). Endophytes can be easily isolated on any microbial or plant growth, such as agar, potato dextrose agar and any nitrogen- or carbon-containing media. The most frequent method used to detect and enumerate endophytes involves isolation from surface-sterilized host plant tissue. The main factors that may regulate entophyte colonization within a plant or microbial species, include the genotype of the plant, the growth stage of the plant, the physiological status of the plant, the type of plant tissues, the environmental condition of the soil in which it is grown, the sampling season, the surface sterility, selective media and culture conditions as well as different agricultural practices (Gaiero et al., 2013; Golinska et al., 2015) Ecological awareness on the role of endophytes in nature, can also provide the best clues for targeting a particular type of endophytic bioactivity with the greatest potential for bioprospecting (Strobel and Daisy, 2003).

## Bioactive Compounds from Endophytes

**FIGURE 1:**
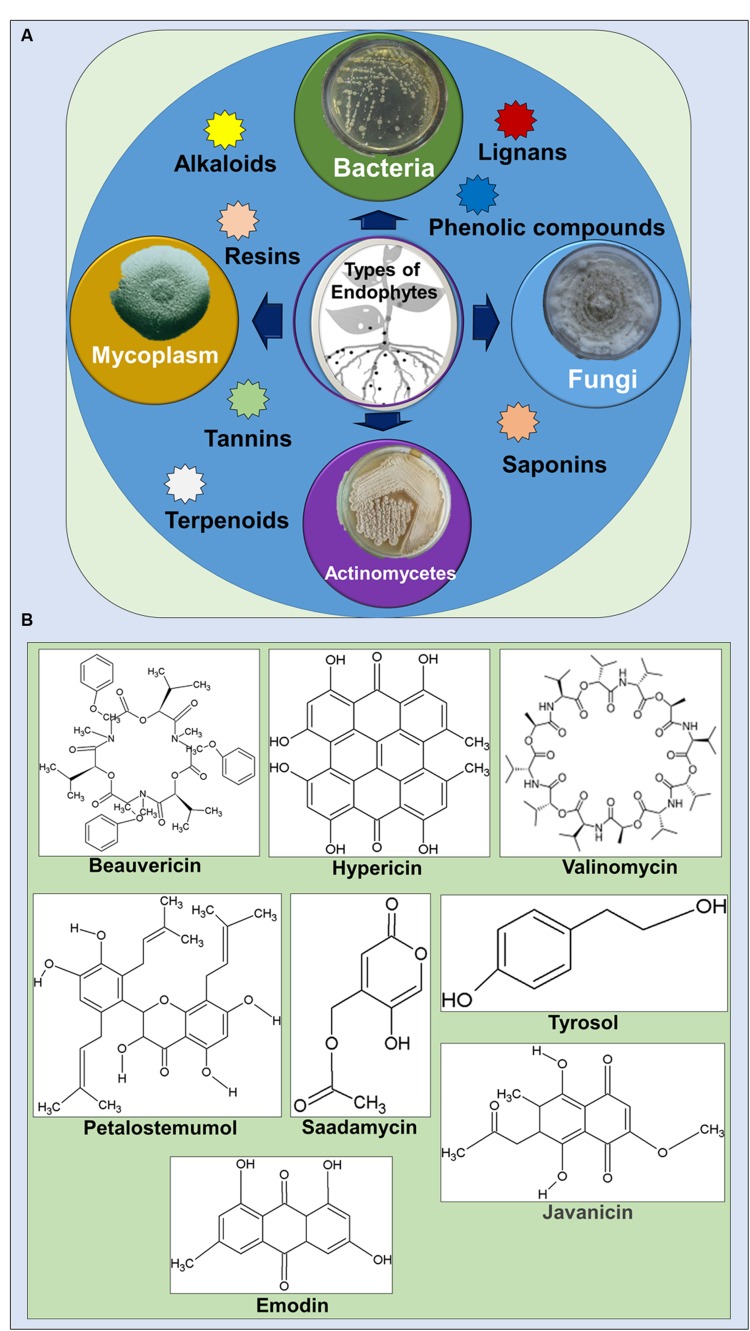
**(A)** Types of endophytes and the bioactive compound groups associated with them. **(B)** Selected bioactive compounds with medicinal importance isolated from endophytes.

Endophytes are reported to produce a number of bioactive metabolites in a single plant or microbe which served as an excellent source of drugs for treatment against various diseases and with potential applications in agriculture, medicine, food and cosmetics industries (Strobel and Daisy, 2003; Jalgaonwala et al., 2011; Godstime et al., 2014; Shukla et al., 2014). These secondary metabolites were categorized into various functional groups, alkaloids, benzopyranones, chinones, flavonoids, phenolic acids, quinones, steroids, saponins, tannins, terpenoids, tetralones, xanthones, and many others (**Figures 1A,B**) (Schulz et al., 2002; Strobel and Daisy, 2003; Jalgaonwala et al., 2011; Joseph and Priya, 2011; Pimentel et al., 2011; Godstime et al., 2014).

Extraction of metabolites from endophytes is affected by various factors, such as the season of sample collection, climatic condition and geographical location (Shukla et al., 2014). However, with a revolutionary synthetic process that has been developed during the past few years, extraction from plants and other natural sources has now become more feasible, efficient and convenient (Hussain et al., 2012). The production of bioactive substances by endophytes, has been directly associated with the evolution of the host microorganisms, which may have incorporated genetic information from higher plants, allowing them to better adapt to the host plant and perform some functions, such as protection from various types of pathogens, insects, and grazing animals (Strobel, 2003). Some of the commonly found secondary bioactive compounds from endophytes are described below.

Taxol (paclitaxol), a complex diterpene alkaloid produced by the endophyte *Metarhizium anisopliae* found in the bark of *Taxus* tree, is one of the most promising anticancer agents developed or synthesized to date (Zhang et al., 2009; Visalakchi and Muthumary, 2010; Jalgaonwala et al., 2011). Camptothecin, from *Nothapodytes foetida* is known to have cytotoxic and antifungal properties (Joseph and Priya, 2011; Han and Rahman, 2012). Huperzine A (HupA), from *Huperzia serrata*, can act as a cholinesterase inhibitor (Nair and Padmavathy, 2014). Lignans, such as cathartics, emetics and cholagogue, isolated from endophytic *Podophyllum hexandrum*, are reported to act as anticancer agents (Konuklugil, 1995). Resins, such as etoposide and teniposide extracted from *P. emodi*, possess strong anticancer activity (Konuklugil, 1995). Compounds such as oxacillin, ampicillin, catechin, gallic acid, and cefalexin are known to possess bactericidal activities (Akiyama et al., 2001). Terpenoids possess antineoplastic, antibacterial, and antiviral effects as well as gastrointestinal stimulation (Jalgaonwala et al., 2011; Godstime et al., 2014). The endophytic fungus, *Cytonaema* sp., produces triterpenoid helvolic acid with strong antibacterial activity (Kumar et al., 2014).

## Microbial Endophytes as Source of Drug Against Various Diseases

Infectious and parasitic diseases account for approximately half of the deaths worldwide (Menpara and Chanda, 2013). Although it is the generation of nano to pico drugs, natural sources have been proven as the best source for drug discovery. Medicinal plants and their endophytes are an important source of precious bioactive compounds and secondary metabolites that contribute to more than 80% of the natural drugs available in the market (Singh and Dubey, 2015). Endophytic microorganisms are the storehouse of novel secondary metabolites that can serve as an excellent source of drugs for antiarthritic, antimicrobial, anticancer, antidiabetic, anti-insect, and immunosuppressant activities (Jalgaonwala et al., 2011; Godstime et al., 2014). To date, only a few plants have been investigated for their endophytic diversity and potential to produce bioactive secondary metabolites. The discovery of novel antimicrobial secondary metabolites and bioactive compounds from different types of endophytic microorganisms is an important alternative to overcome the increasing levels of drugs resistance to various pathogenic microorganisms (Godstime et al., 2014).

There are a number of bioactive compounds, such as camptothecin, diosgenin, hypericin, paclitaxel, podophyllotoxin, and vinblastine, which have been commercially produced by different endophytic fungi present in respective plants and they are of both agricultural as well pharmaceutical importance (Joseph and Priya, 2011; Zhao et al., 2011a). These compounds are analogs of various types of phytohormones, essential oils etc. isolated from various endophytes (Zhao et al., 2011b; Molina et al., 2012; Nicoletti and Fiorentino, 2015). A detailed list of the endophytes isolated from various sources, their bioactive metabolites and the uses of endophytes as a source of medicine against various diseases is presented in **Table 1**.

**Table 1:** Source of bioactive compounds from endophytes and their use against pathogenic microorganisms.

Source of endophytes	Bioactive compounds from endophytes	Cure against pathogen	Mode of transmission of the pathogen	Reference
*Boesenbergia rotunda Streptomyces coelicolor*	Munumbicins	*Escherichia coli*	Ground meats, raw or under pasteurized milk	Golinska et al., 2015 Singh and Dubey, 2015
*Chloridium* sp. *Allamanda cathartica*	Javanicin Munumbicins Phomopsilactone	*Pseudomonas* sp.	Contaminated water or surgical instruments	Jalgaonwala et al., 2011 Nithya and Muthumary, 2011
*Cladosporium* sp.	Cardiac glycosides, phenolic compounds	*Klebsiella pneumoniae*	Contaminated water and aerosols	Selvi and Balagengatharathilagam, 2014
*Cladosporium* sp.	Cardiac glycosides, phenolic compounds	*Proteus* sp.	Canned food products	Selvi and Balagengatharathilagam, 2014
*Cryptosporiopsis quercina*	Saadamycin	*Campylobacter jejuni*	Raw or uncooked poultry and milk	Dutta et al., 2014
*Cytonaema* sp.	Cytonic acids A and B	*Human cytomegalovirus. Hepatitis virus*	Shellfish, berries or contaminated water	Bhardwaj and Agrawal, 2014
*Diaporthe helianthi*	Fabatin, tyrosol	*Enterococcus hirae*	Nosocomial infection through hospitalized patients	Godstime et al., 2014 Specian et al., 2012
*Fusarium proliferatum*	Beauvericin	*Clostridium botulinum*	Improperly processed, canned food	Meca et al., 2010
*Fusarium proliferatum*	Kakadumycin, beauvericin	*Listeria monocytogenes*	Raw or under pasteurized milk, Smoked fish	Golinska et al., 2015 Meca et al., 2010
*Fusarium* sp. *Cryptosporiopsis quercina*	Xularosides, munumbicins, Saadamycin, cryptocandin	*Candida albicans*	Contaminated sweet fruits and milk products	Jalgaonwala et al., 2011 Dutta et al., 2014
*Ganoderma boninense*	Rapamycin, cyclododecane, petalostemumol	*Bacillus subtilis*	Rice, pastas, raw milk and meat products	Parthasarathi et al., 2012; Ismail et al., 2014
*Hypericum perforatum,* *Diaporthe helianthi*	Hypericin, emodin, tyrosol	*Salmonella* sp.	Meat, eggs, and untreated tree nuts	Joseph and Priya, 2011 Specian et al., 2012
*Nigrospora* sp.	Saadamycin	*Fusarium oxysporum*	Maize, cereals, groundnuts and tree nuts	El-Gendy and El-Bondkly, 2010; Dutta et al., 2014
*Phomopsis* sp. *Cinnamomum mollissimum*	Munumbicins, Saadamycin	*Aspergillus niger*	Maize, cereals, groundnuts, and tree nuts	Jalgaonwala et al., 2011 Kaul et al., 2012
*Saccharothrix mutabilis,* *Streptomyces* sp.	Capreomycin Munumbicins	*Mycoplasm* (TB)	Uncooked meat, eggs or poultry	Felnagle et al., 2007 Golinska et al., 2015
*Streptomyces hygroscopicus*	Clethramycin	*Cryptococcus neoformans*	Lettuce harvested from tropical regions	Golinska et al., 2015
*Streptomyces lygroscopicus*	Coronamycin, rapamycin	*Saccharomyces cerevisiae*	Bakery and fermented products	Ezra et al., 2004; Parthasarathi et al., 2012
*Streptomyces* sp.	Kakadumycin A, hypericin	*Shigella* sp.	Contaminated food, water and fecal waste	Golinska et al., 2015 Joseph and Priya, 2011
*Streptomyces* sp. *Achyranthes bidentata,* *Phoma* sp., *Saurauia scaberrinae*	Terephthalic acid Phomodione	*Staphylococcus aureus*	Meat, eggs and dairy products	Hoffman et al., 2008
*Streptomyces* sp., *Kennedia nigricans*	Munumbicins	*Vibrio cholerae*	Raw or undercooked shellfish, particularly oysters	Kumar et al., 2014
*Streptomyces tsusimaensis*	Valinomycin	*Corona virus*	Food or water contaminated with infected fecal matter	Alvin et al., 2014
*Thottea grandiflora*	Streptomyces	*Bacillus cereus*	Uncooked meat and raw milk	Joseph and Priya, 2011
*Xylaria* sp.	dihydroxynaphthol, glucopyranoside	*Herpes virus*	Contaminated body fluid or saliva	Pittayakhajonwut et al., 2005
*Xylaria* sp.	Phenolic compounds	*Streptococcus pyogenes*	Contaminated water, raw milk, salads and eggs	Selvi and Balagengatharathilagam, 2014
*Xylaria* sp. *Ginkgo biloba* *Fusarium proliferatum*	Sordaricin 7 amino-4-methylcoumarin, Beauvericin	*Yersinia enterocolitica*	Swine meat and meat products, milk and dairy products	Joseph and Priya, 2011 Meca et al., 2010

## Recent Developments in the Field of Microbiome Research

Plant roots and the gastrointestinal system of animals play a major role in the absorption of various nutrients and thus harbor a large, complex and dynamic group of microorganisms that help to degrade nutrients to more easily absorbed forms (Ramirez-Puebla et al., 2013). The animal gastrointestinal system is inhibited by a number of microorganisms, starting from the archaea to the eukaryotes along with a number of plant-associated bacteria, particularly the endophytes (Rosenblueth and Martínez-Romero, 2006; Parniske, 2008; Ramirez-Puebla et al., 2013). Similarly, various soil types, moisture, plant genotypes, root lysates etc. are the determinants of the root microbiota (Doornbos et al., 2012).

The diversity of microorganisms in healthy humans vary with diet, maintenance of hygiene, hormonal cycles, infections, uptake of medicine, sexual activity, etc. (Muzny et al., 2013; Gerber, 2014). In this context, various studies associated with the microbiome of humans have been undertaken during recent times. A study from the Human Microbiome Project has also stated the role of the gut microbiome in regulating the host circadian clock, which in mammals is located in the brain (Leone et al., 2015). These studies have provided evidence that a high-fat diet could alter the microbiome circadian rhythm, thereby suggesting a link between the diet, gut microbiota and obesity (Leone et al., 2015).

Furthermore, there are a number of microbiome studies undertaken to find out the associations and diversity of microbiota in both plant and animal systems. Longitudinal microbiome studies are undertaken to gain insights into the dynamic behaviors of the microbiota, such as the microbial succession events during maturation of the infant’s gut, normal temporal inconsistency in healthy adults, responses to various dietary changes and the use of different antibiotics and in instances of dysbiotic alterations that signal symptomatic diseases. Furthermore, the multi-omic analyses that combine information from multiple data sources, such as metabolomes, proteomes and transcriptomes, provide a deep vision into the purposeful changes of the internal microbiome with respect to time. Similarly, the computational tools to analyze microbiome time-series data is another area that shows tremendous growth. These techniques could model the inter-individual variability, while automatically capturing commonalities at appropriate levels in the ecosystems (Gerber, 2014).

## Conclusion

Endophytes are a poorly investigated group of microorganisms capable of synthesizing bioactive compounds that can be used to combat numerous pathogens. These have been dependable sources of bioactive and chemically novel compounds and have proven to be useful for novel drug discovery. Biotransformation methods have a wide range of uses, particularly in the production of numerous bioactive compounds, as antimicrobial (vanillin, essential oils), antifungal and antiviral (alkaloids), antioxidant(eugenol), antiinflammatory (cineole) etc. It is imperative to review and highlight the previous successes, on-going research and latest developments in research associated with endophytic microorganisms to draw the attention of the research community toward this emerging field and possible exploitation of the available sources for their therapeutic uses in various fields, such as the medical, pharmaceutical, food and cosmetics.

## Author Contributions

JKP, SG, GD, and SS wrote the manuscript. H-SS and JKP designed the concept, edited the manuscript. All the authors read and approved the manuscript.

## Conflict of Interest Statement

The authors declare that the research was conducted in the absence of any commercial or financial relationships that could be construed as a potential conflict of interest.
